# Remodelin treatment reshapes inflammation-related transcriptomic signatures in experimental thalamic hemorrhage

**DOI:** 10.3389/fgene.2026.1874675

**Published:** 2026-07-07

**Authors:** Zi Wang, Yaqun Li, Yinggang Xiao, Ju Gao, Tianfeng Huang

**Affiliations:** 1 Department of Anesthesiology, Northern Jiangsu People’s Hospital Affiliated to Yangzhou University, Yangzhou, Jiangsu, China; 2 Yangzhou Key Laboratory of Anesthesiology, Northern Jiangsu People’s Hospital Affiliated to Yangzhou University, Yangzhou, Jiangsu, China

**Keywords:** Cxcl1, immune infiltration, inflammation-related genes, NAT10, neuroinflammation, POMC, Remodelin, RNA sequencing

## Abstract

**Background:**

Thalamic hemorrhage is a severe subtype of intracerebral hemorrhage in which secondary neuroinflammation contributes to tissue injury and neurological deterioration. N-acetyltransferase 10 (NAT10), an RNA N4-acetylcytidine writer, has been implicated in inflammatory regulation and neurological disorders. However, inflammation-related transcriptomic changes associated with Remodelin treatment after thalamic hemorrhage remain unclear**.**

**Methods:**

mRNA transcriptome sequencing was performed on perilesional thalamic tissues from control, thalamic hemorrhage model, and Remodelin-treated mice. Differentially expressed genes were identified for the Model versus Control and Remodelin Intervention versus Model comparisons. Genes showing opposite directions of regulation across the two comparisons were defined as Remodelin-reversed differentially expressed genes. GeneCards-derived inflammation-related genes were converted to mouse orthologs and intersected with Remodelin-reversed genes. Protein-protein interaction analysis, Gene Ontology and KEGG enrichment analyses, gene set enrichment analysis, immune-cell signature estimation, and transcription factor/miRNA regulatory network prediction were performed. Key candidates were validated by quantitative RT-PCR.

**Results:**

RNA-seq identified 499 differentially expressed genes in the Model versus Control comparison and 664 in the Remodelin Intervention versus Model comparison. Among 46 shared differentially expressed genes, 42 showed opposite-direction regulation. Ortholog-corrected screening identified 9 Remodelin-reversed inflammation-related candidates: *Cxcl1*, *Ccl2*, *Ncf4*, *Ptx3*, *Pomc*, *Masp2*, *Tnfrsf8*, *Card9*, and *Gpr84*. Enrichment analyses linked these genes mainly to leukocyte- and neutrophil-mediated immunity, tumor necrosis factor production, cytokine-cytokine receptor interaction, IL-17 signaling, TNF signaling, chemokine signaling, and NOD-like receptor signaling. Protein-protein interaction analysis highlighted *Ccl2*, *Cxcl1*, and *Pomc* as prominent candidate nodes. qRT-PCR using independent biological samples provided preliminary transcript-level support for Remodelin-associated changes in *Cxcl1* and *Pomc* expression, whereas *Ccl2* was increased after hemorrhage but was not significantly reduced by Remodelin.

**Conclusion:**

This study identifies Remodelin-reversed inflammation-related transcriptomic signatures in experimental thalamic hemorrhage. *Cxcl1* and *Pomc* represent the most consistently supported Remodelin-responsive transcriptomic candidates, whereas *Ccl2* appears to be a hemorrhage-associated inflammatory hub without robust reversal at the examined time point. These findings provide an exploratory neurogenomic framework for investigating inflammation-related transcriptional remodeling associated with Remodelin treatment.

## 1 Background

Thalamic hemorrhage refers to the rupture of blood vessels in the thalamic region around the ventricles of the brain. It is characterized by sudden onset headache, impaired consciousness and neurological symptoms. Studies have shown that thalamic hemorrhage accounts for 10%–15% of cases of intracerebral hemorrhage (ICH) and frequently extends into the ventricles, thus resulting in a high mortality and disability rate (Steinke et al., 1992; Qureshi et al., 2009). Patients with hypertension, vascular diseases such as atherosclerosis, or advanced age are more susceptible to thalamic hemorrhage. Patients commonly suffer from cognitive dysfunctions such as aphasia, unilateral neglect, and memory impairments, as well as motor paralysis and sensory disturbances. These deficits can greatly affect a patient’s ability to perform activities of daily living (Maeshima et al., 1997). Due to its deep anatomical location, thalamic hemorrhage is difficult to evacuate (Chen et al., 2021). At present, the ideal treatment for thalamic hemorrhage has yet to be identified. It is necessary to explore new methods to treat this disease.

Beyond the initial mass effect and mechanical tissue disruption, ICH induces rapid transcriptional, epigenetic, and epitranscriptomic reprogramming in resident neural cells and infiltrating immune cells. These regulatory processes may determine the magnitude and duration of post-hemorrhagic inflammation and therefore represent potential therapeutic targets. N-acetyltransferase 10 (NAT10) is the only established mammalian writer of N4-acetylcytidine (ac4C), an RNA modification that can enhance mRNA stability and translational efficiency (Arango et al., 2018; Achour and Oberdoerffer, 2024). NAT10/ac4C dysregulation has been linked to viral RNA stability, cardiovascular injury, and neurological disorders (Tsai et al., 2020; Wang et al., 2022; Guo et al., 2022; Ma et al., 2022). These observations support further investigation of NAT10-relevant RNA regulation in hemorrhagic brain injury.

Inflammatory mechanisms play important roles in ICH. ICH triggers local and systemic inflammatory responses involving resident glial cells and recruited leukocytes; these responses contribute to edema, blood-brain barrier disruption, and secondary neuronal injury (Keep et al., 2012; Chen et al., 2020). NAT10 knockdown has been reported to reduce LPS-induced inflammatory signaling in macrophages (Zhang et al., 2023). Remodelin was originally identified as a NAT10-targeting small molecule (Larrieu et al., 2014), motivating evaluation of Remodelin-associated inflammatory transcriptional changes after thalamic hemorrhage.

In the present study, we screened inflammation-related candidate genes based on transcriptomic data and PPI analysis. We subsequently performed pathway enrichment, computational immune-cell signature estimation, and regulatory network prediction. These analyses were designed to generate hypotheses regarding Remodelin-associated inflammatory transcriptional remodeling after thalamic hemorrhage.

## 2 Methods

### 2.1 Sample collection

All animal experiments were approved by the Animal Care and Use Committee of Yangzhou University (Approval No. 202303874). All methods were carried out in accordance with relevant guidelines and regulations, including the National Institutes of Health Guide for the Care and Use of Laboratory Animals. All experimental protocols were approved by the Animal Care and Use Committee of Yangzhou University. This study is reported in accordance with ARRIVE guidelines (https://arriveguidelines.org) for the reporting of animal experiments.

In this study, transcriptome data from C57BL/6 mice with thalamic hemorrhage were obtained via mRNA transcriptome sequencing. Samples were assigned into a control group, model group (thalamic hemorrhage model), and intervention group (Remodelin treatment), with six samples in each group. Additionally, 1,024 inflammation-related genes (IRGs) were retrieved from the GeneCards database (https://www.genecards.org/) (Stelzer et al., 2016).

### 2.2 Thalamic hemorrhage model induction

Male C57BL/6 mice aged 8–10 weeks and weighing 20–25 g were used in this study. Animals were housed under standard laboratory conditions with a 12-h light/dark cycle and free access to food and water. Mice were randomly assigned to the control, model, and Remodelin intervention groups using a random-number table. 1nvestigators responsible for data analysis were blinded to group allocation.

Mice were anesthetized using vaporized isoflurane (2.5% for induction and 2.0% for maintenance) and positioned in a stereotaxic frame for precise surgical procedures. Collagenase IV (Coll IV; 0.01 U/10 nL), dissolved in sterile saline, was stereotactically injected into the right ventral posterior medial and ventral posterior lateral nuclei of the thalamus to induce thalamic hemorrhage. The injection coordinates relative to bregma were AP 0.82–2.30 mm, ML 1.30–1.95 mm lateral to the midline, and DV 3.01–4.25 mm ventral to the skull surface. The total injection volume was 10 nL, corresponding to 0.01 U collagenase IV per mouse, and the injection was performed at a rate of 10 nL/min to minimize tissue disruption and reflux. Control mice received an equal volume of sterile saline at the same stereotaxic coordinates.

After injection, the glass micropipette was left in place for 10 min to allow complete diffusion of collagenase IV and to minimize reflux. The micropipette was then carefully withdrawn, the cranial opening was sealed with bone wax, and the scalp was sutured with 4–0 surgical sutures.

Mice in the Remodelin intervention group received intraperitoneal injection of Remodelin, a commonly used NAT10-targeting compound, at 5 mg/kg beginning on the first day after stereotaxic injection. Remodelin was first dissolved in dimethyl sulfoxide (DMSO) and then diluted with sterile saline immediately before administration. The final DMSO concentration in the injection solution was 0.014%. Remodelin treatment was administered twice weekly for a total of six injections. The last Remodelin or vehicle injection was administered approximately 72 h before tissue collection. Mice in the model group received the corresponding vehicle solution containing 0.014% DMSO without Remodelin on the same schedule as the Remodelin-treated group. Control mice received stereotaxic sterile saline injection and systemic sterile saline injections on the same schedule.

Predefined exclusion criteria included death before the planned endpoint, severe postoperative complications, failed or misplaced stereotaxic injection, absence of visible hematoma in the intended thalamic region, and RNA samples that did not meet sequencing quality-control criteria. No animals died before the planned endpoint, and no animals were excluded from the final analysis. Perilesional thalamic tissues were collected after completion of the treatment schedule for RNA extraction and subsequent transcriptomic analysis.

### 2.3 Tissue collection and euthanasia

On day 21 post-hemorrhage, approximately 72 h after the last Remodelin or vehicle injection, mice were deeply anesthetized with an overdose of pentobarbital sodium (150 mg/kg, intraperitoneal injection) to ensure humane euthanasia. The absence of heartbeat and respiration was confirmed before tissue collection. The brain was rapidly removed, and the thalamic tissue surrounding the hematoma was carefully dissected on ice for RNA extraction. All procedures were designed to minimize animal suffering and distress.

### 2.4 Transcriptome sequencing

Total RNA was isolated and purified using TRIzol (Invitrogen, CA, United States). RNA quantity and purity were assessed using a NanoDrop ND-1000 spectrophotometer (NanoDrop, Wilmington, DE, United States), and RNA integrity was evaluated using an Agilent Bioanalyzer 2,100 and agarose gel electrophoresis. RNA samples with a concentration >50 ng/μL, RIN >7.0, OD260/280 > 1.8, and total RNA amount >1 μg were used for library preparation. Polyadenylated mRNA was captured using oligo (dT) magnetic beads, fragmented, and reverse-transcribed into cDNA. After second-strand synthesis, end repair, A-tailing, adapter ligation, size selection, and purification, libraries with insert sizes of approximately 300 ± 50 bp were generated and sequenced on an Illumina NovaSeq 6,000 platform.

The retained raw sequencing quality-control table and library quality-control table were reviewed to summarize sequencing and library QC metrics. Across the 18 RNA-seq libraries, the average raw sequencing output was approximately 47.36 million raw reads per sample, corresponding to an average of 7.10 Gb raw bases per sample. The average Q20 and Q30 values were 97.09% and 94.81%, respectively. All 18 libraries passed library quality control, with library fragment sizes ranging from 313 to 344 bp and molar concentrations ranging from 6.86 to 95.8 nM. The archived project files retained raw-data QC metrics and downstream bioinformatics outputs but did not contain complete upstream alignment metadata, including the reference genome build, aligner, gene-level quantification tool, mapping rate, uniquely mapped rate, or complete alignment logs. Therefore, only verifiable computational parameters are reported in this study.

### 2.5 Identification of differentially expressed genes (DEGs) between the control and model groups and between the model and intervention groups

Differential expression analysis was performed using the DESeq2 package in R (Love et al., 2014). Benjamini–Hochberg adjusted P values were calculated for all genes to account for multiple testing. For the primary exploratory screening, genes with nominal p < 0.05 and |log2FoldChange| > 0.5 were defined as differentially expressed genes. This nominal P-value threshold was used because the present study was designed as a candidate-generating transcriptomic analysis with a limited sample size, and an FDR <0.05 threshold at the initial screening stage would have been overly stringent for identifying potentially treatment-responsive genes. To improve biological specificity, downstream analyses focused on genes showing opposite-direction regulation between the Model versus Control and Intervention versus Model comparisons. All nominal P values and Benjamini–Hochberg adjusted P values for the selected genes are provided in Supplementary Table S1. Functional enrichment analyses were performed using the clusterProfiler package, and adjusted P < 0.05 was used as the significance threshold for GO and KEGG enrichment results (Yu et al., 2012).

### 2.6 Screening of key genes

The shared genes were intersected with IRGs to obtain differentially expressed inflammation-related genes (DE-IRGs). Protein-protein association analysis was performed using the STRING database (https://string-db.org) with an interaction score threshold >0.15 (Szklarczyk et al., 2023). Networks were visualized in Cytoscape (Shannon et al., 2003), and densely connected modules were identified using the MCODE plug-in (Bader and Hogue, 2003).

### 2.7 Gene set enrichment analysis (GSEA)

Based on the m2. cp.v2023.1. m.symbols.gmt reference gene set, GSEA was performed using clusterProfiler (Subramanian et al., 2005; Yu et al., 2012). For each candidate gene and comparison (Control versus Model and Model versus Intervention), samples were divided into high- and low-expression groups using the median expression value. Genome-wide differential statistics were calculated using limma (Ritchie et al., 2015), and genes were ranked by log2 fold change before GSEA. Enrichment results with adjusted P < 0.05 were considered significant, and the top five pathways were visualized using enrichplot.

### 2.8 Immune infiltration analysis

To explore associations between candidate genes and computationally inferred immune-cell signatures, the estimated abundance of 25 immune-cell types was calculated from transcriptomic data using CIBERSORT (Newman et al., 2015). Group differences were assessed using the Kruskal–Wallis test. Immune-cell signatures with zero estimates in at least 30% of samples were excluded before visualization. Correlation analyses were then performed between candidate-gene expression and the remaining immune-cell signatures.

### 2.9 Construction of key gene regulatory networks

Transcription factor-target relationships were obtained from the TRRUST v2 database (Han et al., 2018), whereas miRNA-target interactions were predicted using miRNet 2.0 (Chang et al., 2020). The resulting transcription factor-gene and miRNA-gene networks were visualized using Cytoscape (Shannon et al., 2003).

### 2.10 Total RNA preparation and quantitative RT-PCR

qRT-PCR validation was performed using independent biological RNA samples from the same experimental groups rather than the same RNA samples used for RNA-seq; the analysis included n = 3 independent biological samples per group, with three technical replicates per biological sample.

RNA extraction and qRT-PCR were performed based on our previously published protocols (Huang et al., 2020; Fu et al., 2021). Briefly, thalamic tissue was rapidly collected and total RNA was extracted using a Qiagen kit. RNA was reverse-transcribed using ThermoScript reverse transcriptase (Invitrogen) and oligo (dT) primers. qRT-PCR was performed in 20 μL reactions using SYBR Green Supermix (Bio-Rad). Primer sequences were as follows: *Pomc*, 5′-CCT​TTC​CGC​GAC​AGA​GAC​TA-3′ (forward) and 5′-CGT​ACT​TCC​GGG​GGT​TTT​CA-3′ (reverse); *Ccl2*, 5′-AGG​TGT​CCC​AAA​GAA​GCT​GT-3′ (forward) and 5′-AAG​ACC​TTA​GGG​CAG​ATG​CAG-3′ (reverse); *Cxcl1*, 5′-TGG​CTG​GGA​TTC​ACC​TCA​AG-3′ (forward) and 5′-CCG​TTA​CTT​GGG​GAC​ACC​TT-3′ (reverse); and *Gapdh*, 5′-CCT​TCC​GTG​TTC​CTA​CCC​C-3′ (forward) and 5′-GCC​CAA​GAT​GCC​CTT​CAG​T-3′ (reverse). The cycling conditions were 95 °C for 3 min, followed by 40 cycles of 95 °C for 10 s, 60 °C for 30 s, and 72 °C for 30 s. Relative mRNA expression was calculated using the 2^−ΔΔCt method and normalized to *Gapdh*.

Complete archived qRT-PCR assay-validation records, including primer amplification efficiencies, standard-curve R^2^ values, melt-curve output files, no-template controls, no-reverse-transcription controls, and formal reference-gene stability testing, were not available for retrospective reporting. Therefore, the qRT-PCR findings are interpreted as preliminary transcript-level support rather than definitive validation. Future studies will prospectively follow MIQE-consistent assay-validation and reporting procedures (Bustin et al., 2009).

## 3 Results

### 3.1 Remodelin reverses a subset of hemorrhage-associated transcriptomic alterations in the mouse thalamus

**FIGURE 1:**
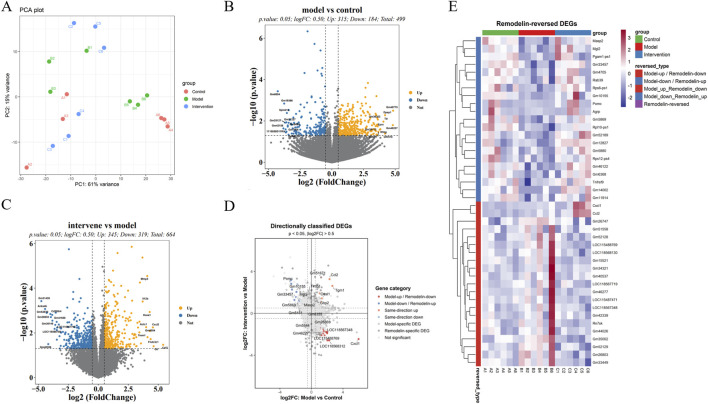
Transcriptomic landscape and Remodelin-reversed gene signatures in the mouse thalamic hemorrhage model **(A)** Principal component analysis (PCA) of RNA-seq samples from the Control, Model, and Remodelin Intervention groups. PC1 and PC2 explained 61% and 15% of the total variance, respectively **(B)** Volcano plot showing differentially expressed genes (DEGs) in the Model versus Control comparison. Red dots indicate upregulated genes, blue dots indicate downregulated genes, and gray dots indicate non-significant genes **(C)** Volcano plot showing DEGs in the Intervention versus Model comparison **(D)** Directional log2 fold-change scatter plot integrating the Model versus Control and Intervention versus Model comparisons. Genes were classified according to their direction of regulation across the two contrasts. Genes upregulated in the Model group and downregulated after Remodelin intervention, or downregulated in the Model group and upregulated after Remodelin intervention, were defined as Remodelin-reversed DEGs **(E)** Heatmap showing the expression patterns of Remodelin-reversed DEGs across the Control, Model, and Intervention groups. Rows represent genes and columns represent samples. Gene expression values were row-scaled. The top annotation indicates sample group, and the side annotation indicates the reversed regulation pattern.

To characterize global transcriptional changes after thalamic hemorrhage and Remodelin intervention, we first performed principal component analysis of the RNA-seq dataset. The control, model, and intervention groups showed visible separation in the PCA plot, with PC1 and PC2 explaining 61% and 15% of the total variance, respectively, indicating that both hemorrhage induction and Remodelin treatment were associated with broad transcriptomic remodeling (Figure 1A). DESeq2 analysis identified 499 DEGs in the model versus control comparison, including 315 upregulated and 184 downregulated genes, suggesting substantial transcriptional disturbance after thalamic hemorrhage (Figure 1B). In the intervention versus model comparison, 664 DEGs were identified, including 345 upregulated and 319 downregulated genes, indicating that Remodelin treatment also induced marked transcriptional changes in the hemorrhagic thalamus (Figure 1C). To distinguish genes that were merely shared between the two contrasts from those showing treatment-associated reversal, we further integrated the direction of log2 fold changes from the model versus control and intervention versus model comparisons. Genes that were upregulated in the model group and downregulated after Remodelin intervention, or downregulated in the model group and upregulated after intervention, were defined as Remodelin-reversed DEGs. Among the 46 shared DEGs, 42 displayed opposite-direction regulation between the two comparisons and were retained as Remodelin-reversed DEGs for subsequent analyses. The full list of RNA-seq-derived Remodelin-reversed DEGs and Remodelin-reversed DE-IRGs, including log2FC values, nominal P values, Benjamini–Hochberg adjusted P values, regulation patterns, and inflammation-related annotation status, is provided in Supplementary Table S1. This directional classification suggests that Remodelin partially counteracted hemorrhage-associated transcriptional abnormalities rather than simply inducing unidirectional gene suppression (Figure 1D). Heatmap visualization of Remodelin-reversed DEGs showed two major expression patterns. One cluster contained genes elevated in the model group and reduced after Remodelin intervention, whereas the other contained genes suppressed in the model group and restored after intervention (Figure 1E). Together, these results demonstrate that thalamic hemorrhage induces prominent transcriptomic dysregulation and that Remodelin directionally modulates a subset of disease-associated genes, providing a foundation for subsequent identification of inflammation-related Remodelin-responsive genes.

### 3.2 Ortholog-corrected screening identified Remodelin-reversed inflammation-related genes

**FIGURE 2:**
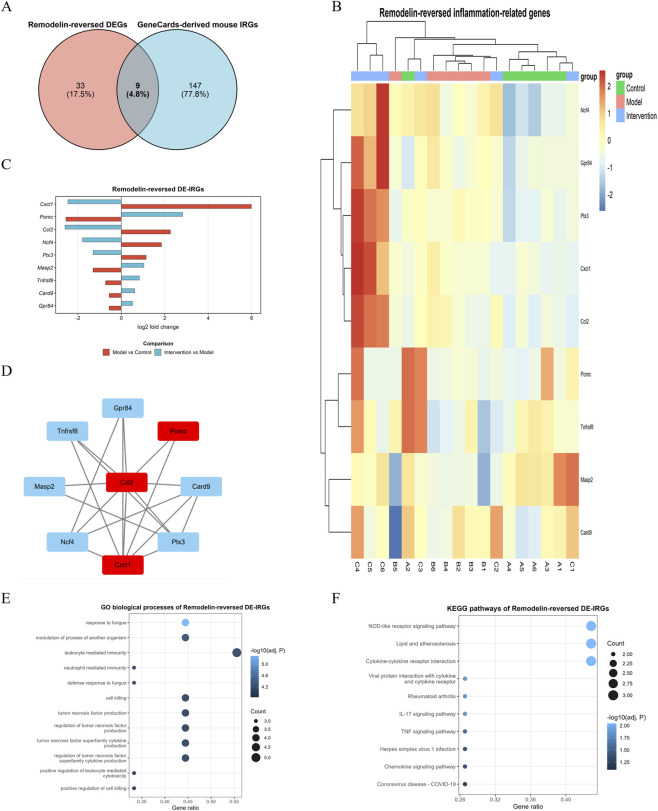
Ortholog-corrected identification and functional characterization of RNA-seq-derived Remodelin-reversed inflammation-related genes **(A)** Venn diagram showing the overlap between RNA-seq-derived Remodelin-reversed DEGs and ortholog-corrected GeneCards-derived mouse inflammation-related genes **(B)** Heatmap showing the expression patterns of the 9 RNA-seq-derived Remodelin-reversed DE-IRG candidates across the Control, Model, and Intervention groups. Rows represent genes and columns represent samples. Expression values were row-scaled. The top annotation indicates sample group **(C)** Dual log2 fold-change barplot showing the direction of regulation of the 9 RNA-seq-derived Remodelin-reversed DE-IRG candidates in the Model versus Control and Intervention versus Model comparisons. Red bars indicate Model versus Control log2FC, and blue bars indicate Intervention versus Model log2FC. Ccl2 showed an opposite-direction pattern in RNA-seq but was not validated as significantly reversed by qRT-PCR **(D)** Protein-protein interaction network of the 9 RNA-seq-derived Remodelin-reversed DE-IRG candidates. Red nodes indicate key candidate genes, including Ccl2, Cxcl1, and Pomc, and blue nodes indicate other inflammation-related reversed genes **(E)** GO biological process enrichment analysis of the Remodelin-reversed DE-IRGs. Dot size represents gene count, and color indicates −log10 adjusted P value **(F)** KEGG pathway enrichment analysis of the Remodelin-reversed DE-IRGs. Dot size represents gene count, and color indicates −log10 adjusted P value.

To identify inflammation-related genes showing treatment-associated reversal, GeneCards-derived human inflammation-related genes were first converted to mouse orthologs and then intersected with RNA-seq-derived Remodelin-reversed DEGs. Venn analysis identified nine overlapping genes, which were defined as RNA-seq-derived Remodelin-reversed DE-IRG candidates (Figure 2A). These genes included *Cxcl1*, *Ccl2*, *Ncf4*, *Ptx3*, *Pomc*, *Masp2*, *Tnfrsf8*, *Card9*, and *Gpr84*. Heatmap analysis showed that these nine candidate genes displayed distinct expression patterns across the Control, Model, and Intervention groups (Figure 2B). Several genes, including *Cxcl1*, *Ccl2*, *Ncf4*, and *Ptx3*, showed increased expression in the Model group and lower expression after Remodelin intervention in the RNA-seq dataset, whereas *Pomc*, *Masp2*, *Tnfrsf8*, *Card9*, and *Gpr84* showed the opposite pattern, with reduced expression in the Model group and increased expression after intervention. These results suggest that Remodelin treatment was associated with directionally altered inflammatory transcriptional modules after thalamic hemorrhage. To further visualize the directionality of these candidates, we plotted log2 fold changes from the Model versus Control and Intervention versus Model comparisons. The dual log2FC plot showed that *Cxcl1*, *Ccl2*, *Ncf4*, and *Ptx3* were upregulated in the Model group and downregulated after Remodelin intervention at the RNA-seq level, whereas *Pomc*, *Masp2*, *Tnfrsf8*, *Card9*, and *Gpr84* were downregulated in the Model group and upregulated after intervention (Figure 2C). Among these genes, *Cxcl1* showed the strongest model-induced upregulation and intervention-associated reduction, while *Pomc* exhibited a clear Model-down/Intervention-up pattern. Protein-protein interaction analysis further revealed a connected interaction network among the 9 RNA-seq-derived Remodelin-reversed DE-IRG candidates, with *Ccl2*, *Cxcl1*, and *Pomc* positioned as prominent candidate nodes within the network (Figure 2D). Functional enrichment analysis indicated that these genes were mainly associated with immune-inflammatory biological processes, including leukocyte mediated immunity, neutrophil mediated immunity, cell killing, tumor necrosis factor production, and regulation of tumor necrosis factor production (Figure 2E). KEGG pathway analysis further linked these genes to NOD-like receptor signaling pathway, cytokine-cytokine receptor interaction, IL-17 signaling pathway, TNF signaling pathway, and chemokine signaling pathway (Figure 2F). Collectively, these results indicate that the RNA-seq-derived Remodelin-reversed DE-IRG candidates are primarily involved in leukocyte/neutrophil-mediated immunity, cytokine and chemokine signaling, TNF-related inflammatory responses, and innate immune pathways.

### 3.3 Key genes play important roles in pathways associated with immune inflammation

**FIGURE 3:**
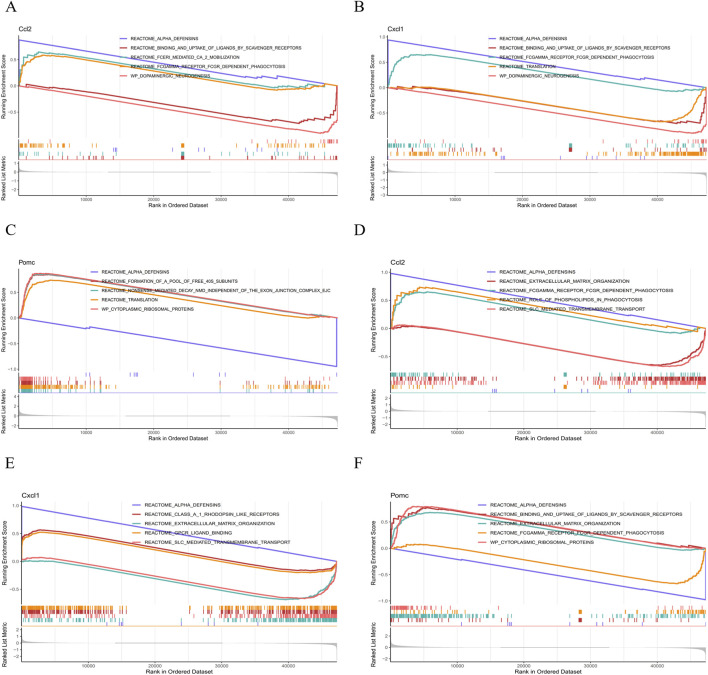
Gene Set Enrichment Analysis (GSEA) enrichment analysis of key genes **(A–C)** Enrichment analysis of key genes in the control group versus the model group **(D–F)** Enrichment analysis of key genes in the model group versus the intervention group.

According to the results of GSEA enrichment analysis of the control group vs. model group, *Ccl2* was enriched in signaling pathways such as alpha defensins and binding and uptake of ligands by scavenger receptors, while *Cxcl1* was enriched in signaling pathways such as alpha defensins and binding and uptake of ligands by scavenger receptors. *Pomc* plays an important role in the signaling pathway of alpha defensins and the formation of a pool of free 40 S subunits (Figures 3A–C). Similarly, in the model group vs. intervention group, *Ccl2* was enriched in signaling pathways that included alpha-defensins and extracellular matrix organization. *Cxcl1* was found to be involved mainly in alpha-defensins. In addition, the *Pomc*-enriched signaling pathways included alpha-defensins and the binding and uptake of ligands by scavenger receptors (Figures 3D–F). These results showed that the signaling pathways enriched in key genes were related mainly to immune inflammation.

### 3.4 Immune infiltration and regulatory network analyses of key inflammation-related genes

**FIGURE 4:**
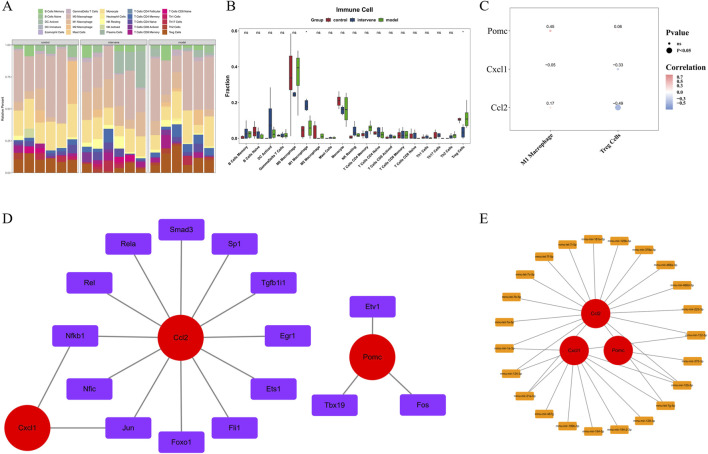
Immune infiltration and regulatory network analyses of key inflammation-related genes **(A)** Estimated abundance of 25 immune cell types across the Control, Model, and Remodelin Intervention groups **(B)** Differentially altered immune cell populations among the three groups **(C)** Correlation analysis between key genes and representative immune cell populations, showing the association between *Ccl2* expression and Treg abundance **(D)** Predicted transcription factor regulatory network of the key candidate genes, including representative regulatory pairs such as *Cxcl1*-Jun, *Ccl2*-Rel, and *Pomc*-Fos **(E)** Predicted miRNA-target regulatory network of the key candidate genes, including representative pairs such as mmu-let-7f-5p-*Ccl2*, mmu-let-7f-5p-*Cxcl1*, and mmu-mir-375-3p-*Pomc*.

To further explore the immune microenvironment associated with the key inflammation-related genes, we estimated the infiltration abundance of 25 immune cell types across the Control, Model, and Remodelin Intervention groups. The overall distribution of immune cell populations showed group-dependent variation (Figure 4A). Among the analyzed immune cell types, M1 macrophages and regulatory T cells (Tregs) differed significantly among the three groups based on the Kruskal test (Figure 4B). Correlation analysis between key genes and differentially altered immune cell types further showed that *Ccl2* was negatively correlated with Treg abundance (Figure 4C), suggesting a potential association between hemorrhage-related chemokine activation and altered immunoregulatory cell signatures.

We next constructed transcriptional and post-transcriptional regulatory networks for the key candidate genes. Transcription factor prediction identified 25 potential TFs associated with *Cxcl1*, *Ccl2*, and *Pomc*, forming regulatory pairs such as *Cxcl1*-Jun, *Ccl2*-Rel, and *Pomc*-Fos (Figure 4D). In addition, miRNA-target prediction identified 23 potential miRNAs targeting the key genes, including mmu-let-7f-5p-*Ccl2*, mmu-let-7f-5p-*Cxcl1*, and mmu-mir-375-3p-*Pomc* (Figure 4E). These findings suggest that the identified inflammation-related genes may be embedded within immune-cell-associated and TF/miRNA-mediated regulatory networks after thalamic hemorrhage and Remodelin intervention.

### 3.5 Validation of the expression of key genes

**FIGURE 5:**
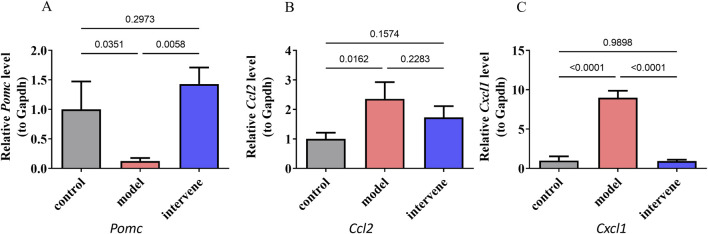
qRT-PCR validation of key candidate gene expression. **(A–C)** qRT-PCR assessment of *Pomc*, *Ccl2*, and *Cxcl1* mRNA expression in the Control, Model, and Remodelin Intervention groups. *Pomc* was downregulated after thalamic hemorrhage and increased after Remodelin intervention, whereas *Cxcl1* was upregulated after thalamic hemorrhage and decreased after Remodelin intervention. *Ccl2* was increased in the Model group but did not show a significant transcript-level reversal after Remodelin treatment. Data are presented as mean ± SEM. One-way ANOVA with Bonferroni *post hoc* correction was used. Exact pairwise P values are shown above the corresponding comparisons. N = 3 independent biological samples per group.

qRT-PCR analysis using independent biological samples provided preliminary transcript-level support for the RNA-seq-derived expression patterns. *Ccl2* and *Cxcl1* were significantly increased in the Model group compared with the Control group, whereas *Pomc* was significantly decreased. After Remodelin intervention, *Pomc* expression increased and *Cxcl1* expression decreased relative to the Model group. However, *Ccl2* expression did not differ significantly between the Model and Intervention groups (Figure 5). These findings support *Cxcl1* and *Pomc* as Remodelin-responsive transcriptomic candidates, whereas *Ccl2* should be interpreted as a hemorrhage-associated inflammatory hub whose RNA-seq-derived reversal was not supported by qRT-PCR at the examined time point.

## 4 Discussion

Thalamic hemorrhage is associated with profound secondary injury driven not only by hematoma formation and mechanical tissue disruption but also by dynamic molecular reprogramming within the perihematomal brain region. Increasing evidence indicates that neuroinflammation, immune-cell recruitment, cytokine signaling, oxidative stress, and transcriptional remodeling collectively contribute to post-hemorrhagic brain injury. In the present study, we investigated the inflammation-related transcriptomic landscape associated with Remodelin treatment in a mouse model of thalamic hemorrhage. By integrating RNA-seq, ortholog-corrected inflammation-related gene screening, protein-protein interaction analysis, pathway enrichment, immune infiltration estimation, regulatory network prediction, and preliminary qRT-PCR assessment, we identified a set of Remodelin-reversed inflammatory gene signatures. Among these candidates, *Cxcl1* and *Pomc* showed the most consistent Remodelin-responsive changes at the transcript level, whereas *Ccl2* appeared to represent a hemorrhage-associated inflammatory hub without robust reversal by Remodelin at the examined time point.

A major conceptual implication of this study is that Remodelin treatment may influence post-hemorrhagic inflammation through transcriptomic remodeling. NAT10 is the only established mammalian writer of N4-acetylcytidine (ac4C), an RNA modification implicated in mRNA stability and translational regulation (Arango et al., 2018; Achour and Oberdoerffer, 2024). Because inflammatory responses after intracerebral hemorrhage require rapid and coordinated changes in cytokines, chemokines, innate immune sensors, and immune-cell activation programs, NAT10-mediated RNA regulation may represent an upstream layer of inflammatory control. Previous work has shown that NAT10 can promote inflammatory activation in macrophages through the NOX2–ROS–NF-κB pathway and that Remodelin, a NAT10 inhibitor, can attenuate inflammatory responses in experimental settings (Zhang et al., 2023). The biological plausibility of a Remodelin–NAT10/ac4C axis in thalamic hemorrhage is further supported by our recent team study, which investigated NAT10 inhibition in experimental thalamic hemorrhage using integrative multi-omics and experimental validation. In that study, NAT10 activity/ac4C modification was assessed by ac4C dot blot, and NAT10 protein expression was evaluated by immunohistochemistry. Thalamic hemorrhage increased ac4C-modified mRNA levels and upregulated NAT10 protein expression, whereas Remodelin treatment reduced ac4C abundance and suppressed NAT10 expression. Behavioral, histological, and molecular evaluations further confirmed that NAT10 inhibition alleviated thalamic hemorrhage-induced damage (Huang et al., 2025).

The ortholog-corrected screening strategy further strengthened the biological relevance of the identified inflammatory signatures. Instead of directly intersecting mouse DEGs with human inflammation-related genes, we first converted GeneCards-derived human inflammation-related genes to their mouse orthologs and then intersected these genes with Remodelin-reversed DEGs. This approach identified 9 RNA-seq-derived Remodelin-reversed DE-IRG candidates, including *Cxcl1*, *Ccl2*, *Ncf4*, *Ptx3*, *Pomc*, *Masp2*, *Tnfrsf8*, *Card9*, and *Gpr84*. Functional enrichment analysis linked these genes to leukocyte-mediated immunity, neutrophil-mediated immunity, tumor necrosis factor production, cytokine-cytokine receptor interaction, IL-17 signaling, TNF signaling, chemokine signaling, and NOD-like receptor signaling. These pathways are highly consistent with known mechanisms of secondary inflammatory injury after intracerebral hemorrhage, in which resident microglia, infiltrating myeloid cells, chemokines, and cytokine networks amplify tissue damage and influence repair (Keep et al., 2012; Chen et al., 2020). Therefore, the RNA-seq-derived Remodelin-reversed DE-IRGs identified here may reflect a Remodelin-associated inflammatory transcriptional module that is biologically compatible with NAT10-sensitive regulation in experimental thalamic hemorrhage.

Among the candidate genes, *Cxcl1* showed a particularly consistent expression pattern, with upregulation after thalamic hemorrhage and downregulation after Remodelin intervention. CXCL1 is a CXC chemokine strongly associated with neutrophil recruitment and early inflammatory cell trafficking. Previous studies have shown that CXCL1/CXCL2 chemokine signaling can control early neutrophil recruitment during tissue inflammation (De Filippo et al., 2013). In the setting of intracerebral hemorrhage, excessive chemokine signaling may contribute to leukocyte infiltration, blood-brain barrier disruption, cytokine amplification, and secondary neuronal injury. The observed reversal of *Cxcl1* expression after Remodelin treatment suggests that Remodelin may attenuate acute chemokine-driven inflammatory recruitment in the hemorrhagic thalamus. However, because this study measured *Cxcl1* at the mRNA level, future studies should confirm whether Remodelin also reduces CXCL1 protein expression and neutrophil infiltration using immunostaining, flow cytometry, or protein-level assays.

*Pomc* also showed a consistent Remodelin-responsive pattern, with decreased expression in the model group and increased expression after Remodelin treatment. POMC is a precursor of multiple melanocortin peptides and is involved in neuroendocrine, metabolic, pain-related, and immune-modulatory processes (Harno et al., 2018). The biological meaning of *Pomc* downregulation in the hemorrhagic thalamus remains to be clarified, but its restoration after Remodelin intervention may indicate partial recovery of neuroimmune or neuroendocrine regulatory programs disrupted by hemorrhagic injury. Melanocortin signaling has been implicated in inflammatory control, and POMC-derived peptides can participate in immune regulation under pathological conditions. Therefore, *Pomc* may represent a candidate link between transcriptomic remodeling and endogenous anti-inflammatory or homeostatic responses after thalamic hemorrhage. Further experiments are needed to determine whether *Pomc* is expressed by specific neuronal or non-neuronal populations in the injured thalamus and whether its restoration contributes functionally to Remodelin-associated neuroprotection.

In contrast, *Ccl2* should be interpreted more cautiously. CCL2, also known as MCP-1, signals through CCR2 and contributes to inflammatory monocyte recruitment after intracerebral hemorrhage (Hammond et al., 2014). In our RNA-seq and network analyses, *Ccl2* showed an opposite-direction expression pattern and occupied a prominent position within the inflammatory gene network. These findings support its role as a hemorrhage-associated inflammatory hub. However, the qRT-PCR assessment suggested that although *Ccl2* was increased after thalamic hemorrhage, its expression was not significantly reduced by Remodelin intervention. Therefore, *Ccl2* should not be overinterpreted as a robust Remodelin-responsive biomarker. Several factors may explain this discrepancy between RNA-seq and qRT-PCR. First, RNA-seq and qRT-PCR differ in sensitivity, normalization strategy, dynamic range, and technical variability. Second, the present study analyzed a single tissue collection time point; *Ccl2* may have a different temporal response window from *Cxcl1* or *Pomc*. Third, *Ccl2* may represent a strong hemorrhage-induced chemokine response that persists despite Remodelin treatment, or it may require a longer intervention period to show significant reversal. Finally, the RNA-seq-derived opposite-direction pattern may reflect biological variability or a false-positive signal under the exploratory DEG threshold. For these reasons, we interpret *Ccl2* as a disease-associated inflammatory hub with incomplete validation as a Remodelin-reversed transcript, whereas *Cxcl1* and *Pomc* are considered the most consistently supported Remodelin-responsive transcriptomic candidates in this study.

The immune infiltration analysis further suggested that Remodelin-associated transcriptomic remodeling may be linked to changes in the immune microenvironment. Among the 25 estimated immune-cell populations, M1 macrophages and regulatory T cells showed significant differences among groups. M1-like macrophages are generally associated with pro-inflammatory cytokine production, oxidative stress, and tissue injury, whereas regulatory T cells are important modulators of immune tolerance, inflammatory resolution, and tissue repair (Mantovani et al., 2004; Arpaia et al., 2015). The negative correlation between *Ccl2* expression and regulatory T-cell abundance suggests a potential relationship between chemokine-driven inflammation and impaired immunoregulatory balance after thalamic hemorrhage. Nevertheless, these results should be regarded as computational estimates derived from bulk RNA-seq rather than direct measurements of immune-cell composition. Validation using immunofluorescence, flow cytometry, or single-cell RNA sequencing would be necessary to confirm whether Remodelin alters macrophage polarization, Treg recruitment, or other immune-cell populations in the perihematomal thalamus.

The predicted transcription factor and miRNA regulatory networks provide additional hypotheses for how Remodelin-reversed inflammatory genes may be regulated. The identified TF-gene pairs, including *Cxcl1*-Jun, *Ccl2*-Rel, and *Pomc*-Fos, are consistent with the involvement of stress-responsive and inflammatory transcriptional programs. JUN, REL/NF-κB family members, and FOS are central regulators of inflammatory and injury-induced gene expression. Similarly, predicted miRNA-gene interactions, including mmu-let-7f-5p-*Ccl2*, mmu-let-7f-5p-*Cxcl1*, and mmu-mir-375-3p-*Pomc*, suggest that post-transcriptional regulation may participate in the inflammatory response after thalamic hemorrhage. Because NAT10 acts at the RNA modification level, these regulatory predictions are particularly relevant. Inflammatory transcript abundance may be shaped not only by transcriptional activation but also by RNA stability, translation efficiency, miRNA regulation, and epitranscriptomic modification. Future studies combining RNA-seq with ac4C-RIP-seq, ribosome profiling, or RNA stability assays could determine whether NAT10 directly modifies transcripts involved in chemokine signaling and inflammatory regulation.

This study has several limitations. First, the analysis was based on bulk RNA-seq of perihematomal thalamic tissue, which cannot resolve cell-type-specific transcriptional changes. The observed gene-expression signatures may reflect changes in neurons, glia, endothelial cells, infiltrating immune cells, or shifts in cell composition. Second, qRT-PCR using independent biological samples provided preliminary transcript-level support for the Remodelin-associated changes in *Cxcl1* and *Pomc*, but protein-level validation was not performed. Complete archived qRT-PCR assay-validation records, including primer amplification efficiencies, standard-curve R^2^ values, melt-curve files, no-template controls, no-reverse-transcription controls, and formal *Gapdh* stability testing, were unavailable for retrospective reporting. Consequently, the qRT-PCR results should not be regarded as definitive validation, and future studies should follow MIQE-consistent procedures (Bustin et al., 2009). Other Remodelin-reversed inflammatory candidates, including *Ptx3* and *Card9*, also require experimental validation. Third, the present study did not directly assess NAT10 protein expression, global ac4C abundance, or ac4C enrichment on the identified inflammatory transcripts. Although our previous experimental work demonstrated increased ac4C-modified mRNA levels and elevated NAT10 protein expression after thalamic hemorrhage, as well as their reduction after Remodelin treatment, the connection between Remodelin treatment and NAT10/ac4C-mediated regulation in the present analysis remains inferential. In particular, *Cxcl1*, *Pomc*, and *Ccl2* should not be interpreted as direct NAT10/ac4C targets without further validation. Fourth, immune-cell signatures were estimated computationally and require validation by immunostaining, flow cytometry, or single-cell analysis. Fifth, the study was performed at a single tissue-collection time point; therefore, the temporal dynamics of chemokine expression, immune-cell infiltration, and tissue repair remain unknown. Finally, functional experiments targeting *Cxcl1*, *Pomc*, or *Ccl2* are required to determine whether these genes are merely associated with Remodelin treatment or are mechanistically involved in its effects after thalamic hemorrhage.

In conclusion, this study identifies a Remodelin-reversed inflammation-related transcriptomic signature in experimental thalamic hemorrhage and provides a neurogenomic framework for understanding how Remodelin treatment may reshape post-hemorrhagic inflammatory programs. *Cxcl1* and *Pomc* emerged as the most consistently supported Remodelin-responsive transcriptomic candidates, whereas *Ccl2* may function as a hemorrhage-associated inflammatory hub without robust transcriptional reversal at the examined time point. These findings generate hypotheses regarding potential NAT10-relevant RNA regulatory mechanisms but do not establish NAT10/ac4C-dependent regulation of the identified transcripts.

## Data Availability

The original contributions presented in this study are publicly available. These data can be found in the Gene Expression Omnibus (GEO) repository with the accession number GSE275066.
